# Supplementary figures and images for: Immunopeptidome profiling of human coronavirus OC43-infected cells identifies CD4 T-cell epitopes specific to seasonal coronaviruses or cross-reactive with SARS-CoV-2
Source: PLoS Pathog. 2023 Jul 27;19(7):e1011032. doi: 10.1371/journal.ppat.1011032 (PMC10409285; doi:10.1371/journal.ppat.1011032)

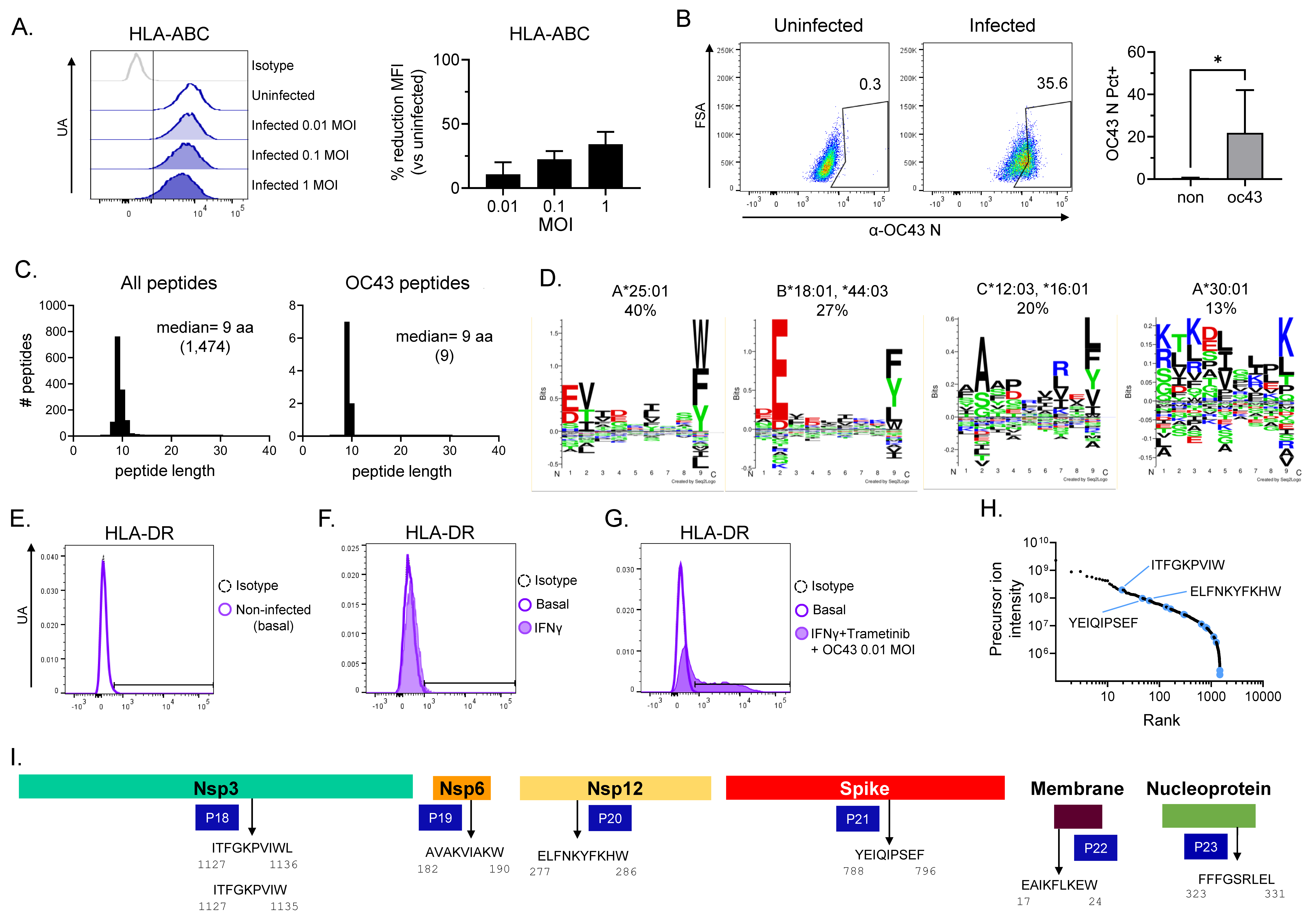

Supplement: S1 Fig — A. HLA-ABC expression on A549 cells uninfected and after the infection with OC43 at different doses; representative histograms and summary of 6 experiments. B. Infection of A549 cells with OC43 at 0.01 MOI. Representative dot plots for intracellular staining for OC43 nucleoprotein (N) in uninfected and infected cells, and summary of 6 experiments. C. Length distribution of the OC43-infected A549 eluted immunopeptidome. D. Sequence logos of clusters obtained using the Gibbs clustering analysis of HLA-ABC eluted immunopeptidome from OC43-infected cells; percentage of peptides in each cluster and probable allele are shown. E. HLA-DR basal expression on uninfected A549 cells. F. Effect of IFN-γ treatment (100 ng/mL) on HLA-DR expression. G. Effect of IFN-γ + trametinib (50 nM) + OC43 infection (0.01 MOI) on HLA-DR expression on A549 cells. H. Eluted peptides ranked by precursor intensity; viral peptides are in blue and top 3 viral peptides sequences are shown. I. Schematic of OC43 source proteins and location of the OC43 eluted peptides. (TIF) [file ppat.1011032.s001.tif]

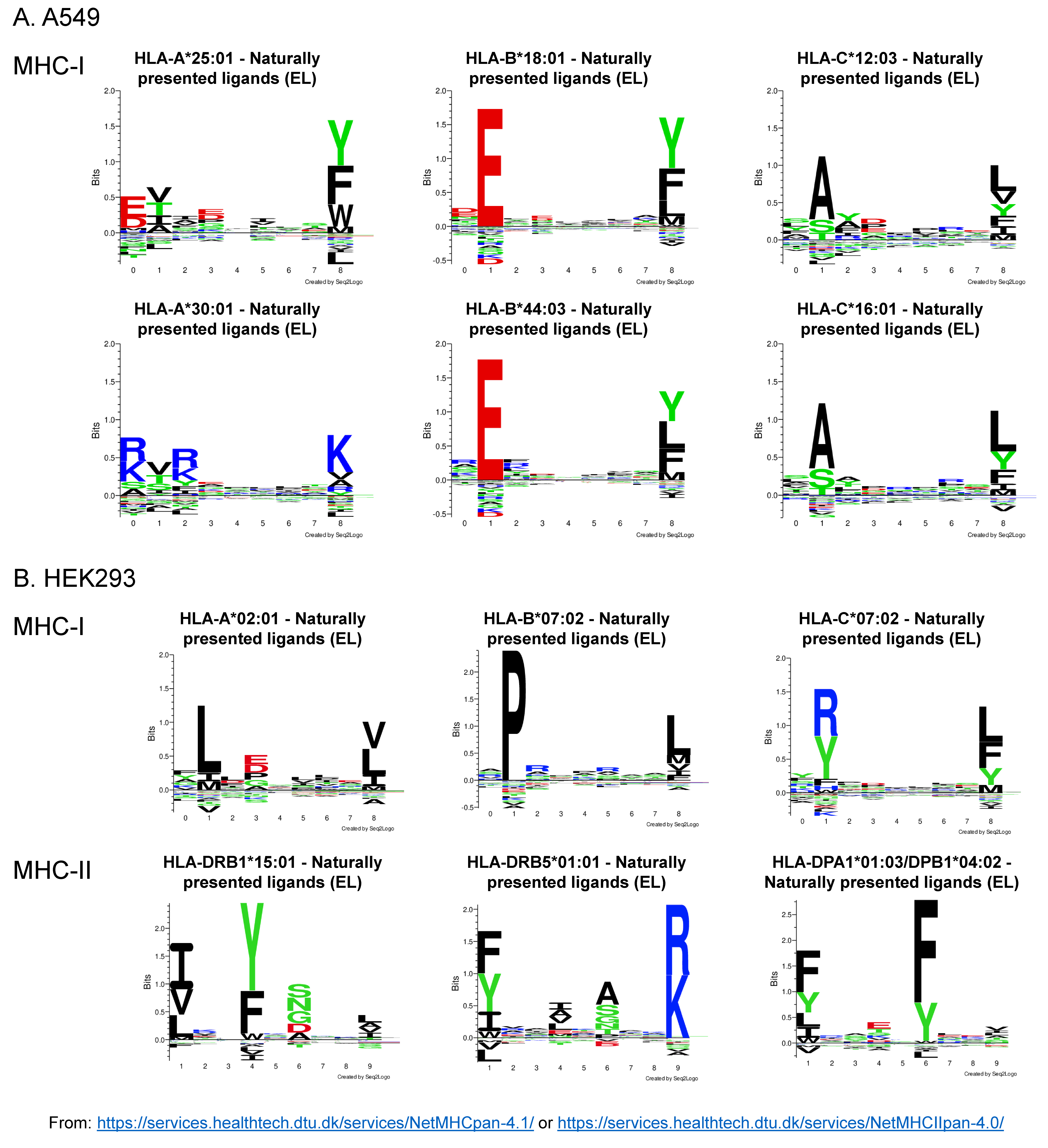

Supplement: S2 Fig — A. MHC-I alleles present in A549 cells. B. MHC-I and MHC-II alleles present in HEK293 cells. Sequence logos of the predicted 9mer core epitope are shown (from Motif Viewer within NetMHCpan 4.1 (https://services.healthtech.dtu.dk/services/NetMHCpan-4.1/) or NetMHCIIpan4.0 (https://services.healthtech.dtu.dk/services/NetMHCIIpan-4.0/), DTU Health Tech). (TIF) [file ppat.1011032.s002.tif]

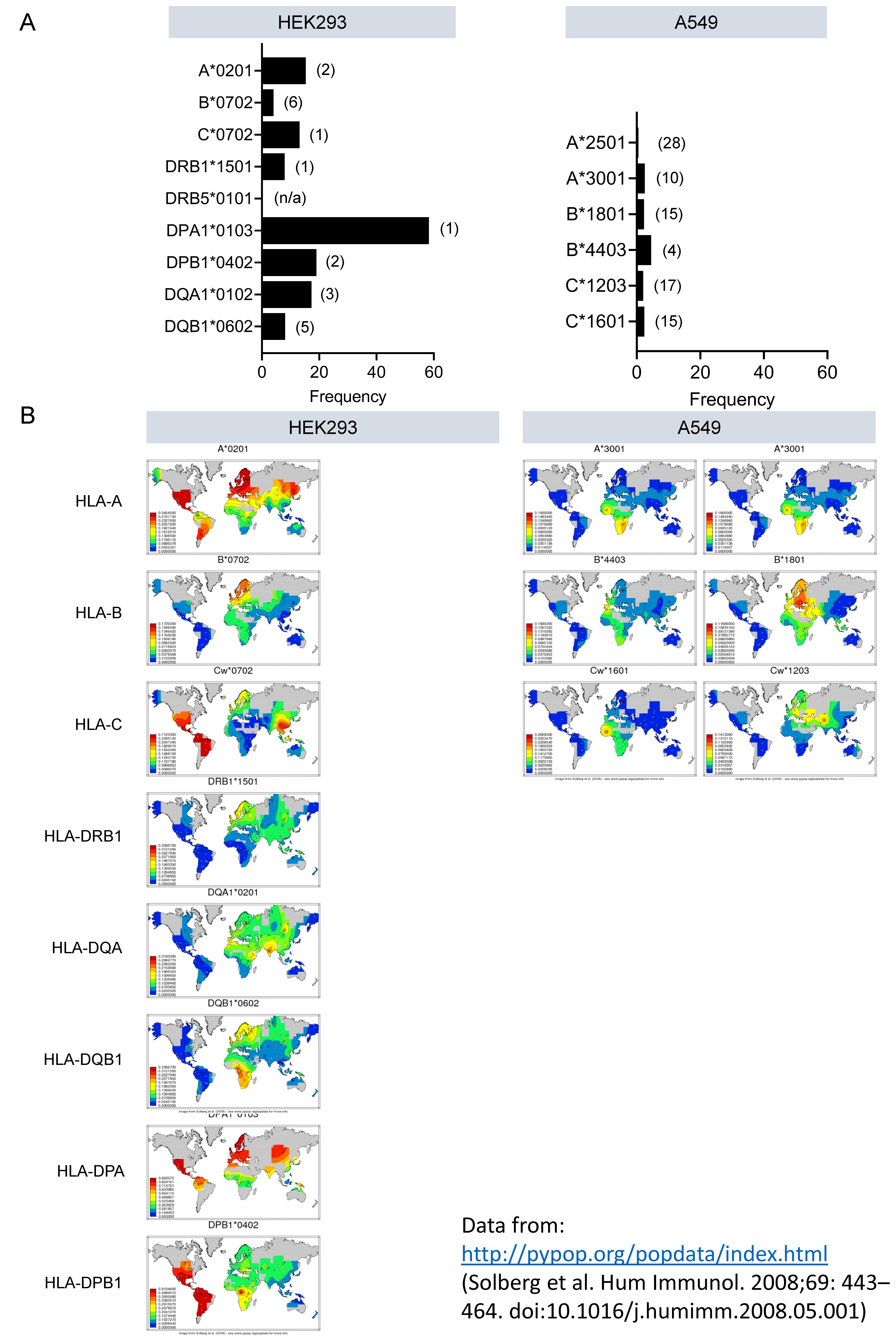

Supplement: S3 Fig — A. Overall average frequency, across all population samples included in Solberg et. al. (2008) [52]; the rank of each allele is shown in parenthesis. B. Geographic interpretations of allele frequency are from a data supplement (http://pypop.org/popdata/index.html) to the article by Solberg et. al. (2008) [52]. (TIF) [file ppat.1011032.s003.tif]

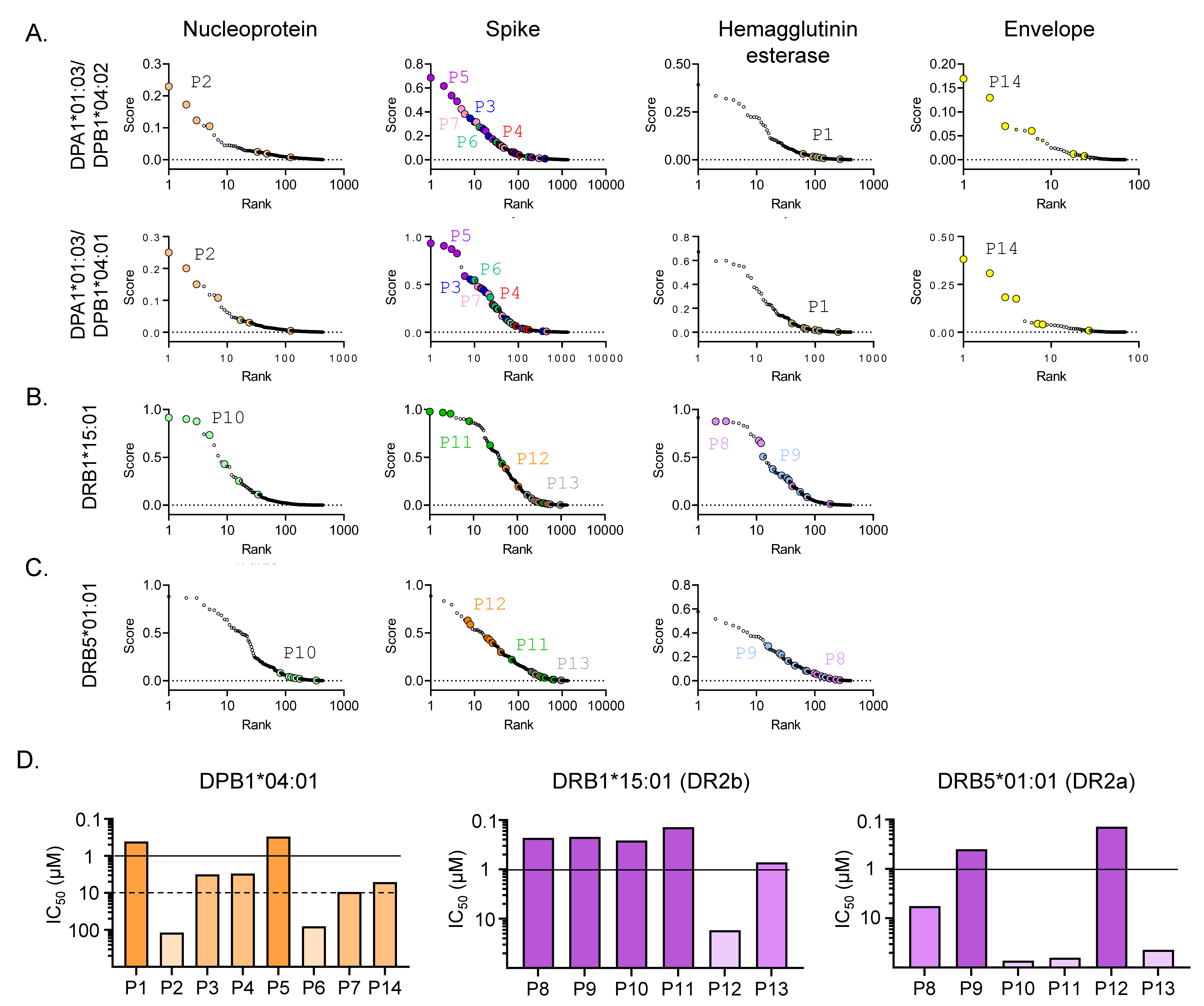

Supplement: S4 Fig — Epitope prediction on whole viral proteins / allele combination were obtained from NetMHCIIpan and sorted by score. Peptides containing the predicted core of the eluted peptides are highlighted in each protein. A. Predictions for DP4.1 and DP4.2; B. Predictions for DR2b; C. Predictions for DR2a. D. Experimental binding of eluted peptides to relevant alleles; dark colors indicate strong binding. (TIF) [file ppat.1011032.s004.tif]

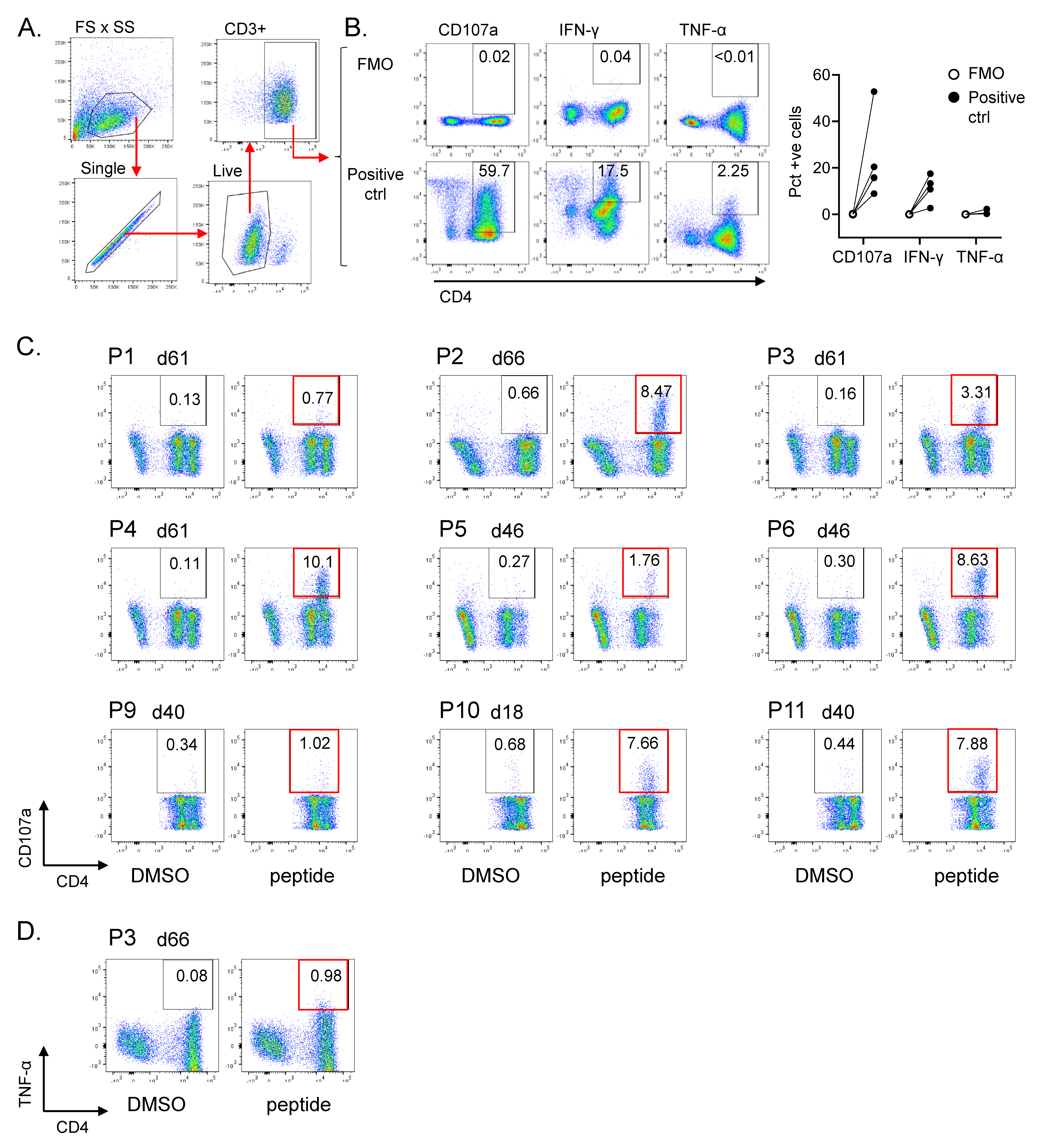

Supplement: S5 Fig — A. Gating strategy for ICS experiments. B. Fluorescence-minus-one (FMO) and positive control (PMA/Ionomycin) representative dot plots and summary in 4 donors. C. CD107a staining of single-peptide in vitro expanded T cells responses to the expanding peptide presented by single allele APC. Dot plots show CD4 (x-axis) and CD107a expression on surface (y-axis). D. ICS for TNF-α production by single-peptide in vitro expanded T cells responses to the expanding peptide presented by single allele APC. Dot plots show CD4 expression (x-axis) and TNF-α production (y-axis). Dot plots for DMSO (background) and peptide are shown. Responses > 3-fold background signal (red boxes) were considered positive. (TIF) [file ppat.1011032.s005.tif]

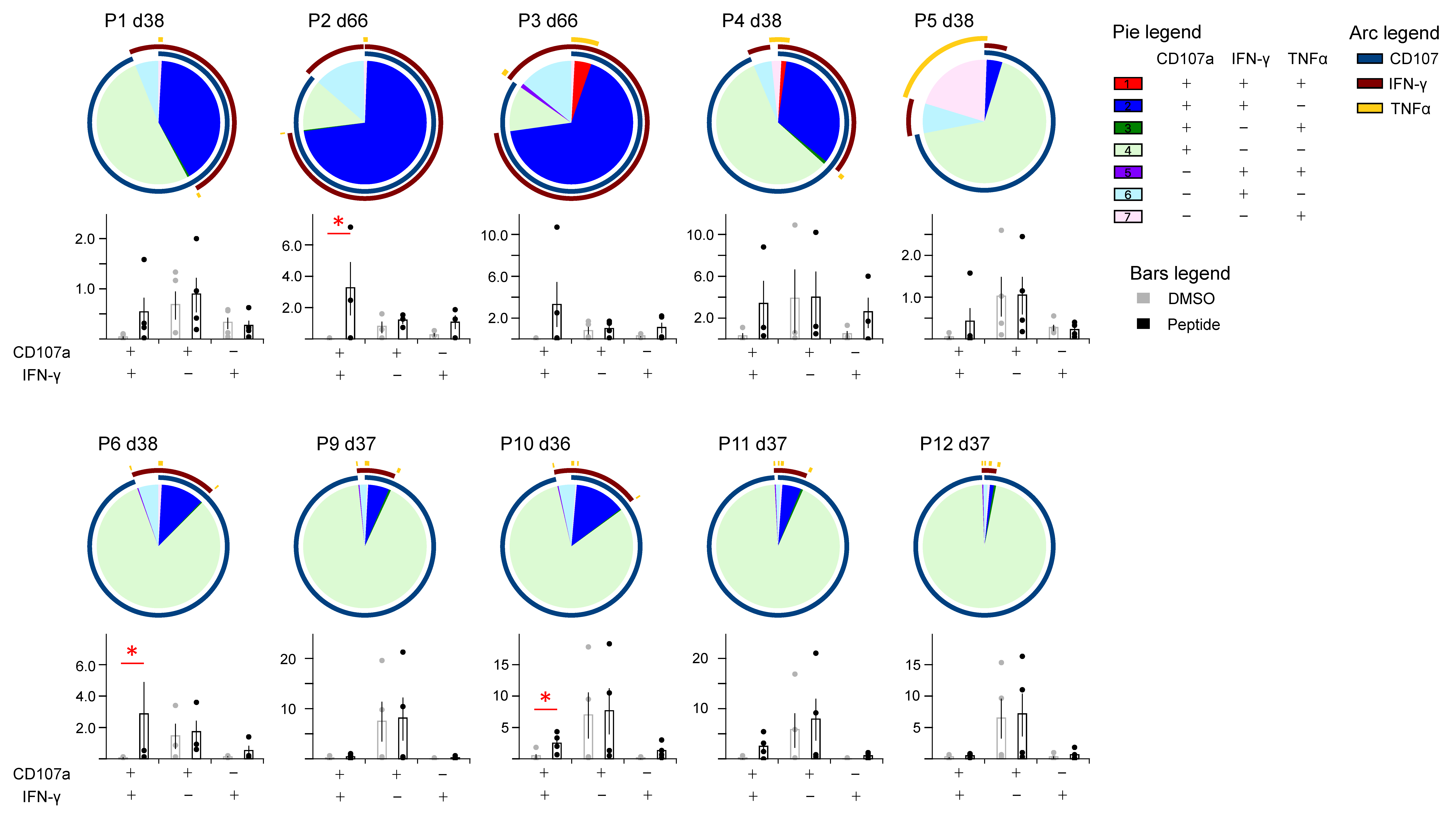

Supplement: S6 Fig — Pie and arc representation of the polyfunctional responses in single-peptide expanded T cells, for individual donors. Bar graphs show a summary of 3–4 donors for each peptide. Boolean gates were exported from FlowJo and analyzed in SPICE [99]. (TIF) [file ppat.1011032.s006.tif]

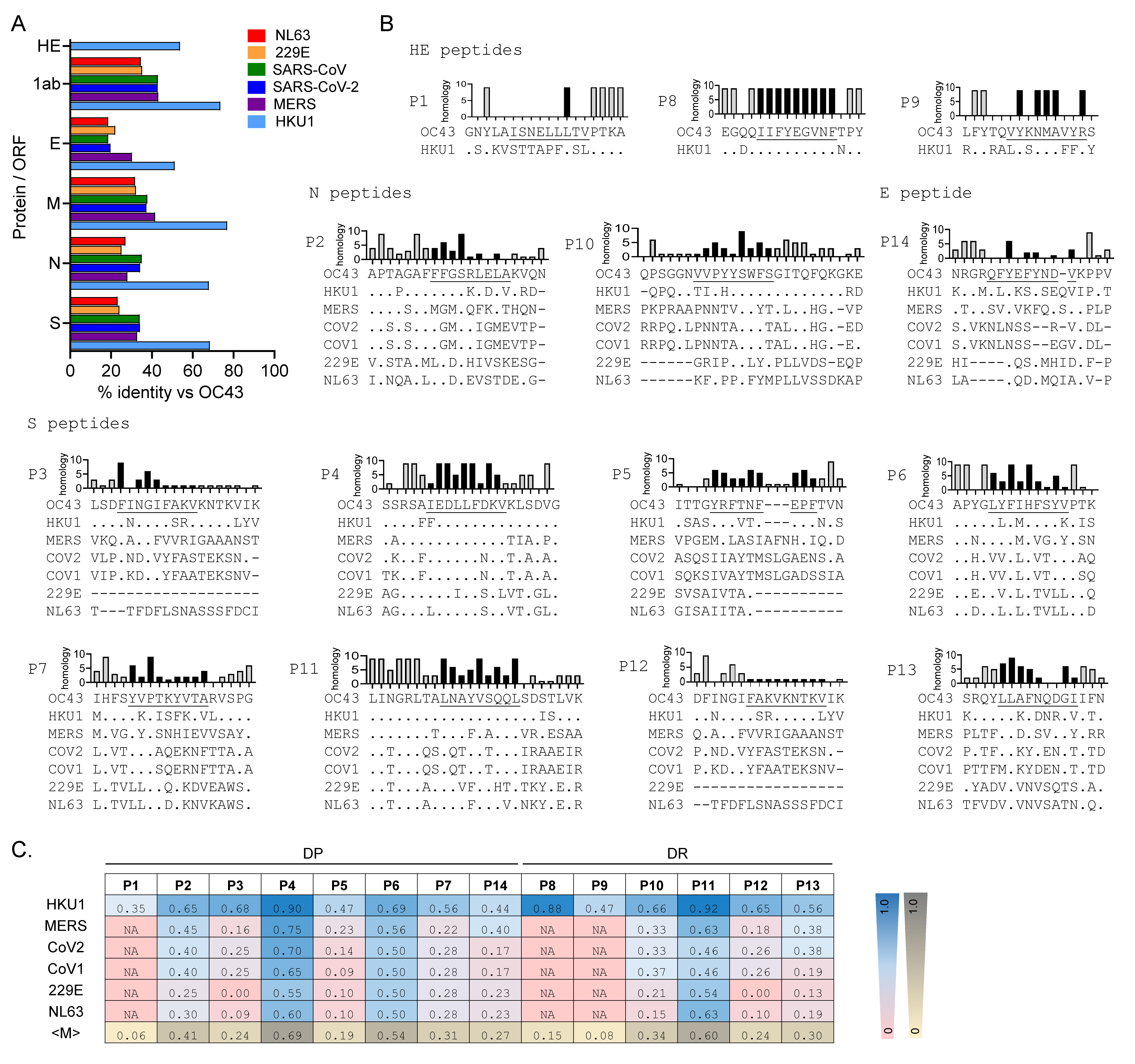

Supplement: S7 Fig — A. Percentage identity of OC43 proteins vs homologous proteins in other human coronaviruses (http://imed.med.ucm.es/Tools/sias.html). B. Sequence alignment of the 14 OC43 eluted peptides to positional homologs in other human coronaviruses. Whole OC43 sequence (with core epitope underlined), and differences in the other sequences are shown. For each alignment, the conservation score at each position was obtained using AL2CO algorithm [101] and presented as a bar graph, with the core epitope positions in black. C. Summary of conservation scores for each eluted peptide to their corresponding homolog peptides in other human coronaviruses. Scores normalized to 100% identity to OC43 peptide as 1, and no conservation as 0. An average per peptide is shown at the bottom of the heatmap. NA indicates no homolog protein between OC43 and the corresponding virus. (TIF) [file ppat.1011032.s007.tif]

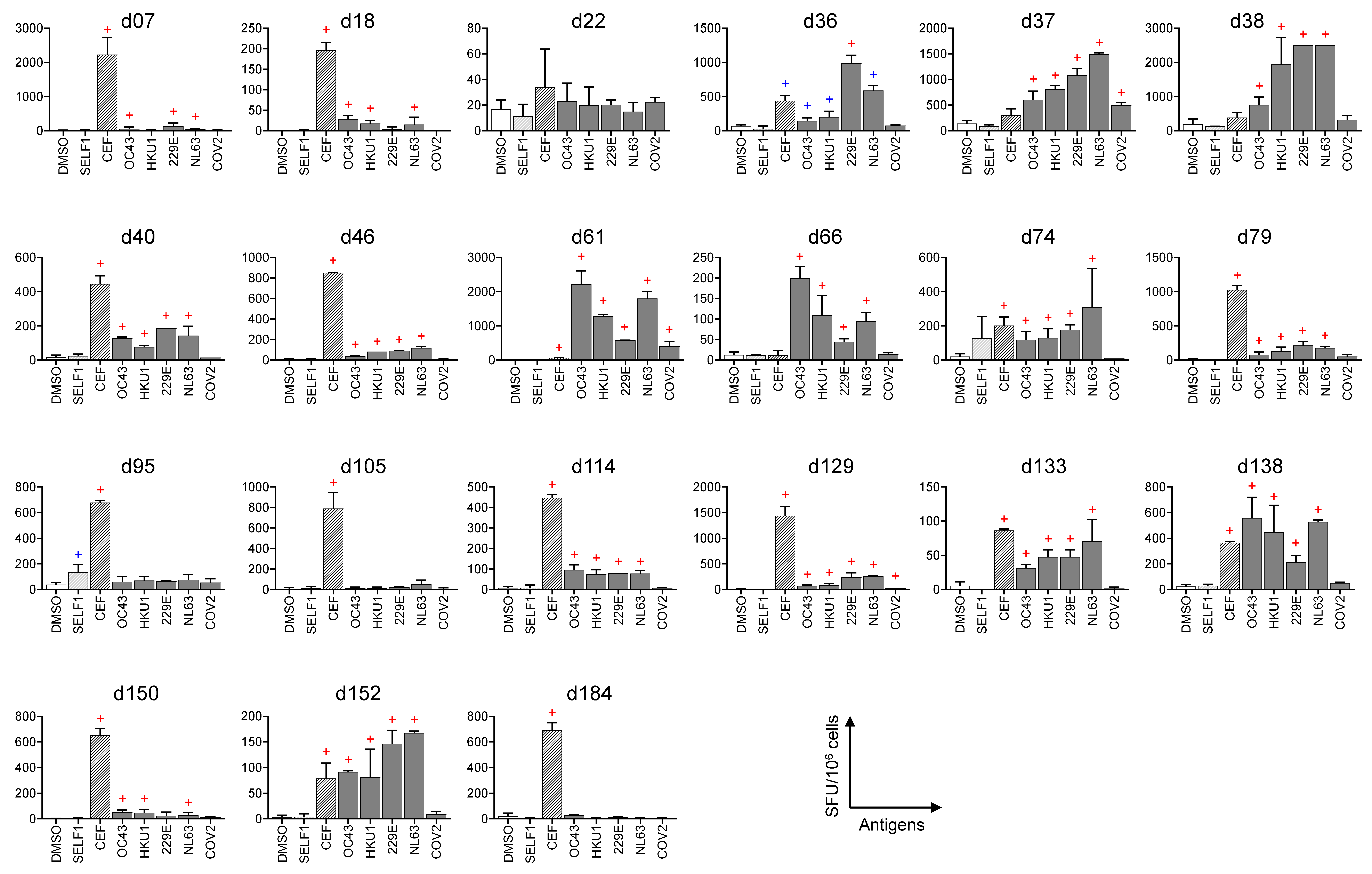

Supplement: S8 Fig — Ex vivo T-cell responses to pools of S protein from OC43, HKU1, 229E, NL63, and SARS-CoV-2 in 21 pre-pandemic donors were measured using IFN-γ ELISpot. DMSO and Self-1 [35] were used as negative controls. Statistical analysis by DFR [98]; positive responses are shown by plus signs (red = DFR2x, blue = DFR1x). (TIF) [file ppat.1011032.s008.tif]
